# *Nlrp12* deficiency alters gut microbiota and ameliorates *Fas^lpr^*-mediated systemic autoimmunity in male mice

**DOI:** 10.3389/fimmu.2023.1120958

**Published:** 2023-03-10

**Authors:** Leila Abdelhamid, Jiangdi Mao, Xavier Cabana-Puig, Jing Zhu, Brianna K. Swartwout, Michael R. Edwards, James C. Testerman, Jacquelyn S. Michaelis, Irving Coy Allen, S. Ansar Ahmed, Xin M. Luo

**Affiliations:** ^1^ Department of Biomedical Sciences and Pathobiology, Virginia-Maryland College of Veterinary Medicine, Virginia Polytechnic Institute and State University, Blacksburg, VA, United States; ^2^ Department of Microbiology, College of Veterinary Medicine, Alexandria University, Alexandria, Egypt; ^3^ Center for Bioinformatics and Computational Biology, University of Maryland, College Park, College Park, MD, United States

**Keywords:** NLRP12, gut microbiota, autoimmunity, sex dependence, pathogenic T cells

## Abstract

NLRP12 has dual roles in shaping inflammation. We hypothesized that NLRP12 would modulate myeloid cells and T cell function to control systemic autoimmunity. Contrary to our hypothesis, the deficiency of *Nlrp12* in autoimmune-prone B6.*Fas^lpr/lpr^* mice ameliorated autoimmunity in males but not females. *Nlrp12* deficiency dampened B cell terminal differentiation, germinal center reaction, and survival of autoreactive B cells leading to decreased production of autoantibodies and reduced renal deposition of IgG and complement C3. In parallel, *Nlrp12* deficiency reduced the expansion of potentially pathogenic T cells, including double-negative T cells and T follicular helper cells. Furthermore, reduced pro-inflammatory innate immunity was observed, where the gene deletion decreased *in-vivo* expansion of splenic macrophages and mitigated *ex-vivo* responses of bone marrow-derived macrophages and dendritic cells to LPS stimulation. Interestingly, *Nlrp12* deficiency altered the diversity and composition of fecal microbiota in both male and female B6/*lpr* mice. Notably, however, *Nlrp12* deficiency significantly modulated small intestinal microbiota only in male mice, suggesting that the sex differences in disease phenotype might be gut microbiota-dependent. Together, these results suggest a potential pathogenic role of NLRP12 in promoting systemic autoimmunity in males. Future studies will investigate sex-based mechanisms through which NLRP12 differentially modulates autoimmune outcomes.

## Introduction

Mice carrying the *Fas^lpr^* mutation are models of autoimmune lymphoproliferative syndrome (ALPS) and systemic lupus erythematosus (SLE) (1). ALPS is a chronic autoimmune disorder characterized by nonmalignant adenopathy and splenomegaly (2), whereas SLE is an autoimmune disease with multisystem involvement (3, 4). Even though the precise etiology for these autoimmune conditions is still unclear, defective apoptosis and expansion of unusual populations of adaptive immune cells (such as double-negative T cells) leading to aberrant lymphoid hyperplasia contribute to the development of autoimmunity in both ALPS (1, 5, 6) and SLE (7, 8). ALPS is primarily a disorder of T cell dysregulation (9–12). SLE, on the other hand, involves a complex interplay between disrupted innate immune functions (13–19) and adaptive immune cell abnormalities (20–26) that contributes to the perturbation of tolerance and development of immunopathogenesis (27). Studies in recent years suggest that microbiota could also modulate autoimmunity and alter disease management outcomes (28, 29). While the role of gut microbial dysbiosis in ALPS remains unknown, dysregulated gut microbiota is a feature of SLE pathogenesis that is known to interact with both innate (30) and adaptive (31) immune responses. We and others have previously unraveled the dynamic changes of gut microbiota in murine lupus and human SLE (32–36) and delineated the influence of gut microbiota modulation on lupus outcomes in different experimental settings (37, 38).

NACHT, LRR and PYD domains-containing protein 12 (NLRP12) is a cytoplasmic innate sensor that plays dual roles in regulating inflammation (39). It is a checkpoint inhibitor controlling inflammation but could also form inflammasome in a context-dependent fashion (39). While the conditions that trigger its regulatory functions are still to be elucidated, NLRP12 has been shown to modulate both innate (40–42) and adaptive (43, 44) immune responses. It is expressed in bone marrow myeloid cells including granulocytes, macrophages and dendritic cells (45) and at a higher level in T cells (43). Interestingly, NLRP12 has been shown to control the activation and migration of myeloid cells (40–42). NLRP12 negatively regulates monocyte/macrophage activation by suppressing the nuclear factor kappa B (NF-κB) signaling (40, 41). Impairment of NLRP12 significantly hinders the migration and responsiveness of dendritic cells (DCs) and neutrophils to chemokine stimulation (42). In parallel, a single missense mutation in *Nlrp12* results in defective neutrophil recruitment (46). In addition, the absence of *Nlrp12* impairs CXCL1 production by macrophages and DCs and subsequently hinders neutrophil recruitment in response to various inflammatory stimuli (46) (47). Moreover, while its role in B cell regulation is still to be determined, NLRP12 could negatively regulate the activation of various T cell subsets including Th1, Th2 and Th17 in a cell-intrinsic manner (43, 44, 48). Importantly, NLRP12 has been shown to regulate immune responses through modulating the gut microbiota (49–51).

The role of NLRP12 under an autoimmune environment is not fully understood. In fact, NLRP12 has controversial roles in modulating organ-specific inflammatory disorders. For instance, it has been shown to play protective roles in colitis (52); meanwhile, it exerts dual roles in modulating brain inflammation in experimental autoimmune encephalitis (EAE, a mouse model of multiple sclerosis) (53, 54). The role of NLRP12 in systemic autoimmune disorders such as ALPS and SLE is unknown. In the current work, we have investigated the role of NLRP12 in a *Fas^lpr^*-mediated autoimmune mouse model of ALPS and SLE, B6/*lpr*. We hypothesize that NLRP12 would modulate myeloid cells and T cells to control inflammation under this autoimmune condition. Surprisingly, our data has shown that the deficiency of *Nlrp12* ameliorates autoimmunity in our model in a sex-dependent manner. To better understand this observation, we have also delineated the cellular mechanisms through which NLRP12 might shape autoimmune pathogenesis. In addition, we concurrently observed the dynamic changes of gut microbiota upon alteration of NLRP12 that may correlate with disease attenuation in male B6/*lpr* mice.

## Materials and methods

### Experimental animals

All experiments were conducted in compliance with the IACUC guidelines of Virginia Tech. *Nlrp12*-deficient B6/*lpr* was generated by cross-breeding B6.*Nlrp12^-/-^* (42) with B6.*Fas^lpr/lpr^* mice (The Jackson Laboratory, Bar Harbor, ME). Offspring were genotyped for both the *Nlrp12* locus and *Fas^lpr^* mutation (**Figure S1**). We monitored the disease progression in both female and male mice housed under specific pathogen-free environment in an AAALAC accredited animal facility at Virginia Tech. All factors including housing, handling, light cycle (12-hour light/dark) were consistent for all mice, which received the hormone-free NIH-31 Modified 6% Mouse/Rat diet. Food and water were provided *ad libitum*.

### Assessment of renal function

The development of lupus nephritis was assessed through weekly testing of proteinuria levels. Weekly urine samples were collected, and proteinuria levels were measured using a Pierce Coomassie Protein Assay Kit (Thermo Scientific) as we previously described (55). Additionally, upon euthanasia at 39 weeks of age, kidneys were harvested to determine the deposition of immune complexes in the renal compartments through immunohistochemical staining for IgG as described below. Renal deposition of complement C3 was also determined with immunohistochemical staining.

### Measurement of serum testosterone

Endpoint serum samples were sent to the Ligand Assay & Analysis Core of the Center for Research in Reproduction (CRR) at the University of Virginia for measurement of testosterone levels. Mouse serum testosterone levels were determined using Testosterone Mouse & Rat ELISA (IBL America) following the manufacturer’s recommendations.

### Cell isolation and *in vitro* stimulation

Total splenocytes and bone marrow (BM) cells were isolated and red blood cell exclusion was achieved following our previously published protocols (55). Both Splenocytes and BM cells were analyzed using flow cytometry as described below. Furthermore, for *in vitro* generation of BM-derived myeloid cells, BM cells from femurs were cultured at a density of 10^6^ cells/ml for 6 days in complete RPMI medium (RPMI 1640 supplemented with 10% fetal bovine serum, 1 mM sodium pyruvate, 1% 100 MEM non-essential amino acids, 10 mM HEPES, 55 μM 2-mercaptoethanol, 2 mM L-glutamine, and 100 U/ml penicillin–streptomycin; all from Life Technologies, Grand Island, NY) supplemented with 10 ng/ml recombinant murine GM-CSF (PeproTech) and cultured for 6 days as previously described (56). For *in vitro* stimulation of BM-derived myeloid cells, cultures were treated with 50 ng/ml or 1 μg/ml lipopolysaccharide (LPS; eBioscience) for four hours before analysis. At the end of the stimulation period, cells were harvested for both flow cytometry and RT-qPCR analysis whereas the supernatants were collected for ELISA.

### Flow cytometry

Cells were initially blocked with anti-mouse CD16/32 (eBioscience) then stained with fluorochrome-conjugated antibodies following our previously published procedures (55). Zombie Aqua (BioLegend) staining was performed to exclude dead cells. For quantification of B cells in total splenocytes, the following anti-mouse antibodies were used: CD19-Pacific blue, CD27-PE, CD138-APC-Cy7, CD44-PerCP-Cy5.5, IgD-PE-Cy7, GL7-AF647. For splenic T cells, CD3-APC, CD4-FITC, CD8-PE, CD44-PerCP-Cy5.5, CD62L-APC-Cy7, CD69-Pacific blue, CXCR5-PerCP-Cy5.5, and PD-1-APC-Cy7 (BioLegend) were used. For myeloid cell analysis, the following anti-mouse antibodies were used: CD11b-PE, CD11c-PerCP-Cy5, F4/80-PE-Cy7 (BioLegend), Gr1-V540 (BD Bioscience). Analysis was performed with a BD FACSAria II flow cytometer (BD Biosciences). Flow cytometry data were analyzed with FlowJo.

### Immunohistochemistry

Spleen and kidney were harvested at the endpoint and embedded in Tissue-Tek OCT Compound (Sakura Finetek) and rapidly frozen in a freezing bath of dry ice and 2-methylbutane. Frozen OCT samples were cryosectioned and unstained slides were stored at −80°C. Immunohistochemical staining procedures were performed as we previously described (55). Splenic sections were stained for germinal center (GC) formation using the following anti-mouse antibodies: CD3-APC, IgD-PE, GL7-FITC (BioLegend). Renal immune complex deposition was determined using anti-IgG-PE (eBioscience) and anti-C3-FITC (Cedarlanelabs, Burlington, Canada). Slides were mounted with Prolong Gold containing DAPI (Life Technologies). Pictures were visualized with both an EVOSVR FL microscope (Advanced Microscopy Group, Grand Island, NY) and a Zeiss LSM 880 confocal microscope (Zeiss,USA, Fralin Imaging Center, Virginia Tech). Image processing and quantification of the fluorescent intensity were performed with ImageJ and ZEN 2.1 Lite software. Sections from at least 3 mice per group were quantified and the unit used for calculation was “integrated density score.”

### RNA extraction and RT-qPCR

Total RNA extraction was performed from snap-frozen pre-weighed splenic tissue or snap-frozen cultured cells as we previously reported (55, 57). Tissues or cell pellets were homogenized in Qiazol lysis reagent using TissuelyserII homogenizer (Qiagen). Total RNA was isolated using RNeasy Plus Universal Kit (Qiagen) with the elimination of gDNA. Reverse transcription (RT) was carried out using iScript™ Reverse Transcription Supermix (Bio-Rad). Quantitative PCR (qPCR) was performed utilizing the Fast SYBR^®^ Green Master mix and the ABI 7500 Fast Real-Time PCR System (Applied Biosystems). Relative transcript quantities were calculated using the 2−ΔΔCt method and normalized to the level of the 18S rRNA housekeeping gene level. Primer sequences for mouse *Bcl6, Prdm1*/Blimp1*, Tnfsf13b*/BAFF*, Il21, Tnf, Il1β, Cxcl13, Ccl19*/MIP-3β*, Ccr7*, and androgen receptor are available in **Table S1**.

### ELISA

Serum samples were obtained at euthanasia, and aliquots were stored at −80°C until processing. Anti-doubles stranded (ds)DNA IgG antibodies were determined following our previously reported procedures (55). Serum BAFF, IL-6 and IFNγ were determined using ProcartaPlex™ Multiplex Immunoassay (Invitrogen) following manufacturer’s procedures and the data were acquired and analyzed using the Luminex FlexMAP3D™ system (Chicago, USA). For culture supernatants, TNFα was determined using mouse TNFα ELISA MAX kit (BioLegend) following manufacturer’s procedures.

### Microbiota sampling and analyses

Fecal microbiota samples from each mouse at the indicated time points were obtained by taking a mouse out of the cage and collecting a fecal pellet. To avoid cross-contamination, each microbiota sample was collected by using a new pair of sterile tweezers. Samples were stored at −80°C. Similarly, at euthanasia, different intestinal sections (duodenum/jejunum, ileum, and colon) were recovered immediately, and the contents of each section were separately collected by manual extrusion and frozen immediately at −80°C until use. All samples were processed at the same time. Sample homogenization, cell lysis, and DNA extraction were performed as previously described (55, 58). For 16S rRNA sequencing, the V4 region (ca. 252 bp) of 16S rRNA gene was PCR amplified with 515F and 12-base GoLay barcoded 806R primers (59). The purified amplicons were sequenced bidirectionally (150 bp PE chemistry) on an Illumina MiSeq at Argonne National Laboratory. Samples were analyzed using the R package phyloseq (60). Reads were processed and amplicon sequence variants (ASVs) were generated using DADA2 in R. Reads were quality trimmed and filtered using the command fastqPairedFilter with parameters truncLen=c(140,140), maxEE=c(2,2), rm.phix=TRUE, maxN=0, compress=TRUE, multithread=FALSE. DADA2 was used to learn error rates, perform sample inference, dereplicate and merge paired-end reads, and construct a sequence table (61). Taxonomy was assigned using the SILVA 138 ribosomal RNA (rRNA) database training set (62) using the DADA2 functions, assignTaxonomy and addSpecies. A total of 3327 ASVs were detected in 212 total samples. ASVs seen fewer than three times in at least 20% of samples and samples with fewer than 1000 reads were removed from the dataset, resulting in 205 samples and 187 ASVs used for downstream analyses. ASVs were aggregated at the genus level using the phyloseq function *tax_glom*. Counts were used for alpha diversity and differential abundance tests, while proportions were used to calculate Bray-Curtis dissimilarity. Differentially abundant and variable taxa between groups were identified using the function *differentialTest* in corncob (63) and significance was assessed using a Wald test with an FDR cutoff of 0.05. Shannon diversity was calculated using the DivNet (64) functions *divnet* and *testDiversity*. Bray-Curtis distances were calculated using the phyloseq function *ordinate*, specifying “method=“NMDS”, distance=“bray”, trymax=1000”. Significance was assessed using the adonis test in the vegan package with 999 permutations.

### Statistical analysis

Student’s *t* test was employed for the comparison between two groups. For *in vitro* culture data involving more than 2 groups, two-way ANOVA with Sidak’s multiple comparison test was employed. Data are shown as mean ± standard error of the mean (SEM). Significant differences were shown as *P < 0.05, **P < 0.01, ***P < 0.001, ****P < 0.0001. All analyses were performed with Prism GraphPad.

## Results

### *Nlrp12* deficiency ameliorates hallmarks of autoimmunity in male B6/*lpr* mice

**Figure 1:**
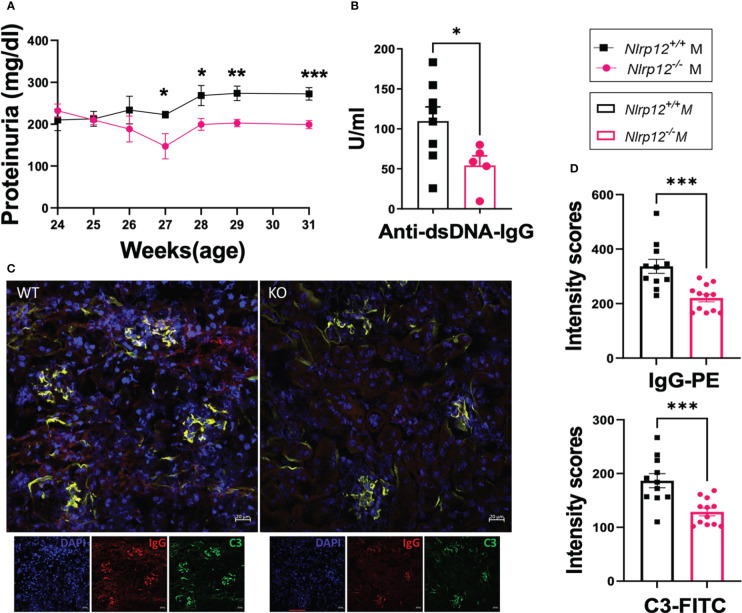
*Nlrp12* deficiency ameliorates hallmarks of autoimmunity in male mice with *Fas^lpr^*-mediated systemic autoimmunity. The progression of systemic autoimmunity in a mouse model of ALPS and SLE was assessed in male *Nlrp12^+/+^* (WT) and *Nlrp12^-/-^* (KO) B6/*lpr* mice. **(A)** Level of proteinuria over time (n=6 or 8/group). **(B–D)** Endpoint analyses at 39 weeks of age. **(B)** Level of anti-double stranded (ds)DNA IgG antibodies. **(C)** Immunohistochemical stains of kidney sections showing the deposition of IgG (red) and C3 (green) with DAPI staining of nuclei (blue). Pictures were captured with a Zeiss LSM 880 confocal microscope. Bar, 20 μm. **(D)** Mean intensity scores of IgG-PE and C3-FITC fluorescence as determined by ZEN 2.1 Lite software. Student’s *t* test was employed for the comparison between two groups. Data are shown as mean ± SEM. Significant differences were shown as *P < 0.05, **P < 0.01, and ***P < 0.001.

Since sex differences exist (65), where females are more generally affected with autoimmune disease (66–68), to investigate the roles of NLRP12 in modulating inflammation in the B6/*lpr* model of autoimmunity, we monitored the disease progression in both male and female mice. Interestingly, the deficiency of *Nlrp12* did not alter disease progression in female mice (**Figures S2A, B**). In contrast, while splenomegaly was not affected (data not shown), the gene deletion significantly mitigated several hallmarks of lupus disease in male B6/*lpr* mice, including reduced proteinuria levels (**Figure 1A**), decreased circulatory levels of anti-dsDNA antibodies (**Figure 1B**), and reduced deposition of IgG and complement C3 in renal compartments (**Figures 1C, D**), indicating sex-specific effects of NLRP12 in modulating lupus pathogenesis. Interestingly, we found a trending increase in both serum testosterone level (**Figure S2C**) and the splenic transcript level of androgen receptor (**Figure S2D**) in male *Nlrp12-/-* B6/*lpr* compared to *Nlrp12+/+* (WT) B6/*lpr* mice, suggesting a potential role for sex hormones.

Notably, we monitored male mice from 24 to 39 weeks of age. WT B6/*lpr* mice generally develop systemic autoimmunity without significant clinical pathology of renal inflammation or nephritis, which was confirmed in our studies. However, *Nlrp12-/-* B6/*lpr* mice exhibited even lower proteinuria levels that were significantly different from WT B6/*lpr* mice during the earlier time window from 24 to 31 weeks of age (**Figure 1A**), while we could not detect differences in proteinuria level during the later period from 32 to 39 weeks of age (**Figure S1E**).

Together, these findings indicate that NLRP12 might have pathological roles in modulating systemic autoimmunity in male B6/*lpr* mice. From now on, we will focus on describing male mice unless noted otherwise.

### *Nlrp12* deficiency dampens B cell activation and differentiation

**Figure 2:**
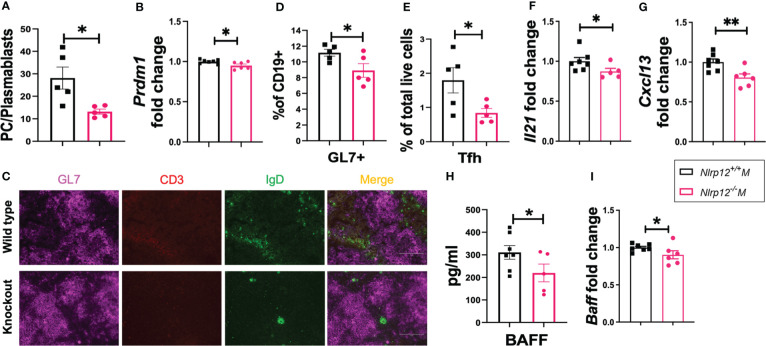
*Nlrp12* deficiency dampens B cell activation and differentiation. Spleens were harvested at the endpoint of 39 weeks of age. **(A)** The ratio of the frequencies of plasma cells *vs*. plasmablasts in total splenocytes. **(B)** Relative transcript level of splenic *Prdm1.* **(C)** Immunohistochemical stains of splenic sections with GL7 (purple), CD3 (red), and IgD (green). Pictures were captured with an EVOSVR FL microscope. **(D)** GL7^+^ cells as a percentage of splenic CD19^+^ B lymphocytes as determined with flow cytometry. **(E)** Percentage of Tfh cells in total splenocytes. **(F)** Relative transcript level of splenic *Il21*. **(G)** Relative transcript level of splenic *Cxcl13*. **(H)** Level of serum BAFF as determined with Luminex assay. **(I)** Relative transcript level of splenic *Tnfsf13b*/BAFF. Student’s *t* test was employed for the comparison between two groups. Data are shown as mean ± SEM. Significant differences were shown as *P < 0.05 and **P < 0.01.

We detected a significantly lower level of autoantibodies and their renal deposition in the absence of NLRP12. Therefore, to delineate the mechanisms through which *Nlrp12* deficiency protects against inflammation in our autoimmune model, we investigated its effects on B cell responses. Deficiency of *Nlrp12* suppressed B cell responses in male B6/*lpr* mice (**Figure 2**). *Nlrp12^-/-^* B6/*lpr* mice had significantly reduced plasma cells (gated as CD19^-^CD27^-^CD138^+^IgD^-^) to plasmablasts (gated as CD19*^+/low^* CD27*^+/low^* CD138^+^IgD^-^) ratio in total splenocytes (**Figure 2A**), suggesting a blockade right before terminal differentiation of B cells. This is consistent with a significant reduction of the splenic transcript level of *Prdm1* (**Figure 2B**). Interestingly, we also found a nearly significant reduction of the transcript levels of the master regulator of the germinal center (GC) reaction, *Bcl6*, in splenic tissues of *Nlrp12-*deficient mice (**Figure S3A**). Although GC formation shown as GL7 staining in immunohistochemically stained splenic sections was not different (**Figure 2C**, **Figure S3B**), there was a significant reduction of the percentage of GL7^+^ cells in total CD19^+^ splenic B lymphocytes (**Figure 2D**; gating strategy is shown in **Figure S3C**). Moreover, we found that *Nlrp12*-deficient mice had a significantly reduced percentage of splenic T follicular helper (Tfh) cells (**Figure 2E**; gated as CXCR5^+^PD-1^+^CD4^+^CD3^+^ in **Figure S3D**), as well as significantly reduced staining of CD3^+^ cells in the GCs (**Figures 2C**, **S3E**). Notably, the percentages of Tfh cells were low and highly variable in the WT mice, and the deficiency of NLRP12 further decreased the frequencies of these cells. Furthermore, while we only found a trending reduction of serum IL-21 (**Figure S3F**), a major cytokine produced by Tfh cells (69), we detected a significant reduction in its splenic transcript level in *Nlrp12*-deficient mice (**Figure 2F**). These results indicate that *Nlrp12* deficiency might dampen GC reaction by suppressing the functions of Tfh cells. Finally, we found downregulated levels of factors assisting B cells (70) including the splenic transcript levels of the B cell chemoattractant *Cxcl13* (**Figure 2G**) and the circulatory level of the B cell survival factor BAFF (**Figure 2H**) as well as its splenic transcript level (**Figure 2I**). These results indicate that *Nlrp12* deficiency dampens terminal differentiation, GC reaction, and survival of potentially autoreactive B cells, which might be the reason for decreased production of autoantibodies and ameliorated autoimmune pathologies. Further studies will elucidate whether NLRP12 targets Bcl-6 and/or Blimp-1 to control autoreactive B cell responses.

### *Nlrp12* deficiency decreases T cell expansion and responses

**Figure 3:**
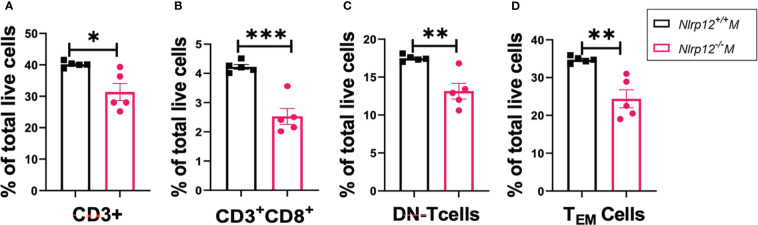
*Nlrp12* deficiency decreases T cell expansion and responses. Spleens were harvested at the endpoint of 39 weeks of age. The percentages of total T **(A)**, CD8^+^ T **(B)**, DN-T **(C)**, and T_EM_ **(D)** cells in total splenocytes as determined with flow cytometry are shown. Data are shown as mean ± SEM. Significant differences were determined by Student’s *t* test and shown as *P < 0.05, **P < 0.01, and ***P < 0.001.

Activation of NLR proteins can shape T cell differentiation and responses. For instance, activation of inflammasome-forming NLRs such as NLRP3 often results in the production of proinflammatory cytokines that could drive the differentiation of inflammatory T cells including Th1 and Th17 (71). However, the exact immunoregulatory functions of NLRP12 in modulating T cell differentiation and responses are still elusive (44, 54). Since T cells play pivotal roles in amplifying and maintaining inflammation particularly through activating autoreactive B cells (72), producing disease-promoting cytokines, and accumulating autoreactive memory (73), we sought to determine how *Nlrp12* deficiency modulates the frequencies and responses of different T cell populations. Deficiency of *Nlrp12* significantly reduced percentage of CD3^+^ T cells in total splenocytes (**Figure 3A**), consistent with the reduced fluorescence intensity of CD3^+^ T cells in immunohistochemically stained splenic GCs (**Figure S3E**). *Nlrp12^-/-^* B6*/lpr* mice also had significantly fewer CD8^+^ (**Figure 3B**) and double negative (DN)-T cell (**Figure 3C**) percentages in total splenocytes, which possibly contributed to the reduction in CD3^+^ T cells. CD4^+^ T cell response did not change. Importantly, the generation of DN-T cells is one of the prominent alterations of T cell responses reported in SLE (8) and ALPS (74–76). These DN-T cells could have been generated from activated CD8^+^ T cells (74, 77–79). Moreover, we found a reduced proportion of CD44^+^CD62L^−^ effector memory T (T_EM_) cells in the spleens of *Nlrp12*-deficient mice (**Figure 3D**). Together, these results suggest that *Nlrp12* deficiency might target T cells to dampen autoimmunity in male B6/*lpr* mice.

### *Nlrp12* deficiency reduces pro-inflammatory macrophage responses

**Figure 4:**
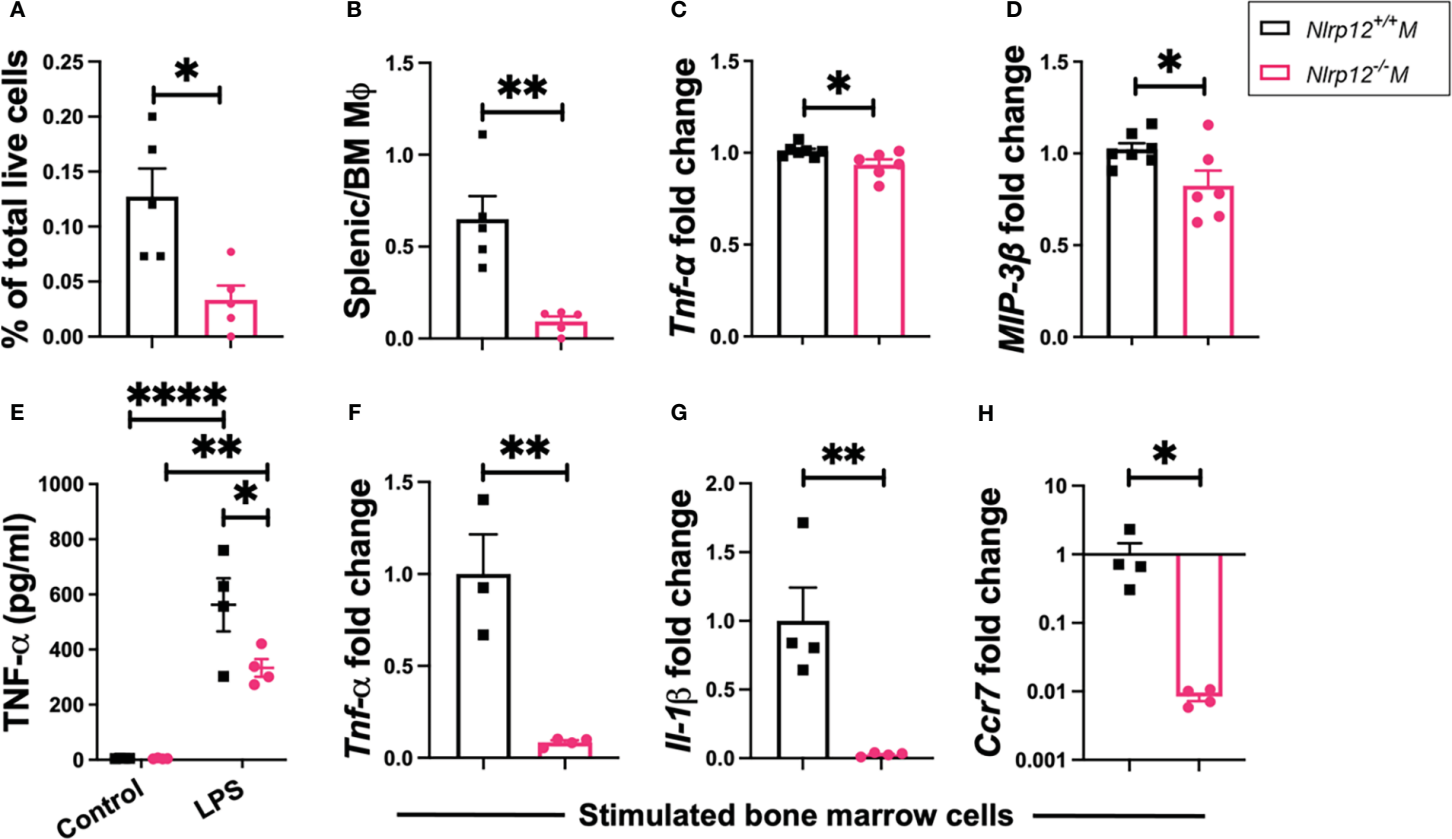
*Nlrp12* deficiency reduces pro-inflammatory macrophage responses. Spleens and BM were harvested at the endpoint of 39 weeks of age. **(A)** The percentage of Gr1^-^F4/80^+^CD11b^+^CD11c^-/low^ macrophages in total splenocytes. **(B)** The ratio of splenic to BM macrophages. **(C)** Relative transcript level of splenic *Tnf.* **(D)** Relative transcript level of splenic *Ccl19*/MIP-3β. **(E-H)** BM cells were stimulated *ex vivo*. **(E)** Level of TNFα in the culture supernatant as determined with ELISA following 4-h stimulation with 1 μg/ml LPS. **(F-H)** Transcript levels of *Tnf***(F)**, *Il1β* **(G)** and *Ccr7* **(H)** as fold changes over unstimulated controls following 4-h stimulation with 50 ng/ml LPS. Data are shown as mean ± SEM. Significant differences were determined by Student’s *t* test (**A–D**, **F–H**) or two-way ANOVA **(E)** and shown as *P < 0.05, **P < 0.01, and ****P < 0.0001.

NLRP12 can modulate the responsiveness of different myeloid cells including neutrophils, dendritic cells (DCs) and macrophages (40–42, 46, 47). We examined the immunophenotypic changes of these populations (see **Figure S4A** for gating strategies) in different lymphoid compartments including BM and spleen. We found no significant changes in neutrophils (**Figure S4B**; gated as CD11c^-^CD11b^+^Gr1^+^) or DCs (**Figures S4C, D**; gated as CD11c^high^ CD11b^+^Gr1^-^ or CD11c^high^ CD11b^+^Gr1^+^). However, *Nlrp12^-/-^* B6*/lpr* mice showed a significant reduction of Gr1^-^F4/80^+^CD11b^+^CD11c^-/low^ macrophages as the percentage of total splenocytes (**Figure 4A**). In addition, as the percentage of BM macrophages slightly increased in *Nlrp12^-/-^* B6/*lpr* mice (**Figure S5A**), the ratio of splenic-to-BM macrophages was significantly reduced with *Nlrp12* deficiency (**Figure 4B**), suggesting decreased migration of these cells from BM to the spleen. Importantly, we also detected significantly reduced splenic transcript levels of *Tnf* (**Figure 4C**) and macrophage inflammatory protein 3-β (MIP-3β, gene name *Ccl19*; **Figure 4D**). These data suggest that *Nlrp12* deficiency might dampen pro-inflammatory responsiveness of splenic macrophages in autoimmune environment. Interestingly, following *ex-vivo* stimulation of BM-derived myeloid cells with LPS – a potent activator of macrophages (80) that could prime DCs (81) – although there were slightly more BM-derived macrophages with *Nlrp12* deficiency regardless of stimulation status (**Figure S5B**), the percentage of BM-derived DCs in these cultures was significantly reduced with the deficiency (**Figure S5C**). This suggests decreased priming of DCs and thus reduced functional potential of BM-derived macrophages. In parallel, we detected a significantly reduced level of TNFα in the culture supernatants of BM-derived cells with *Nlrp12* deficiency following LPS stimulation (**Figure 4E**). Similarly, LPS-stimulated BM-derived cells from *Nlrp12^-/-^* B6/*lpr* mice had reduced transcript levels of *Tnf* (**Figure 4F**, **Figure S5D** following 50 ng/ml and 1 μg/ml LPS stimulation, respectively) and *Il1β* (**Figure 4G**, **Figure S5E**), as well as *Ccr7* (**Figure 4H**, **Figure S5F**), a receptor known to be expressed on DCs following their activation (82). These *ex-vivo* findings suggest a potential pathogenic role of NLRP12 in potentiating macrophages and DCs in response to pro-inflammatory triggers. Importantly, the change of IL-1β suggests that NLRP12 inflammasome may facilitate the production of IL-1β that in turn drives the production of other inflammatory mediators including TNF-α (83) and MIP-3β (84). Together, these results suggest reduced pro-inflammatory innate immunity with *Nlrp12* deficiency in male B6/*lpr* mice.

### *Nlrp12* deficiency induces dynamic changes in gut microbiota diversity and composition

**Figure 5:**
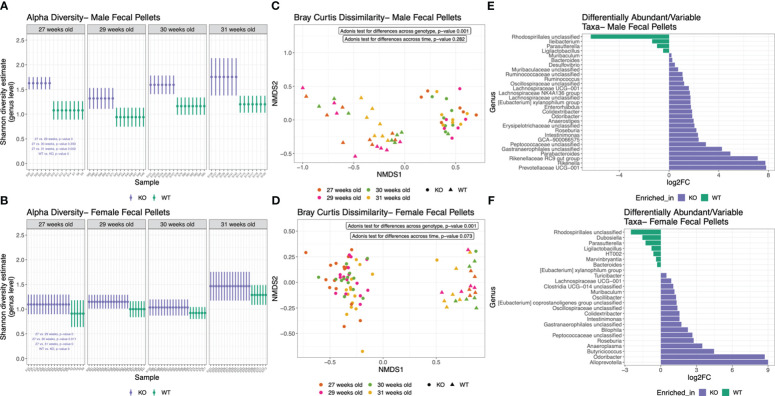
*Nlrp12* deficiency induces dynamic changes in fecal microbiota diversity and composition. **(A, B)** Alpha diversity of fecal microbiota based on Shannon diversity estimate in male **(A)** and female **(B)** B6/*lpr* mice upon alteration of NLRP12. **(C, D)** Non-metric multidimensional scaling (axes NMDS1 vs. NMDS2) showing the segregation of fecal microbiota overtime based on Bray Curtis dissimilarity of beta diversity in male **(C)** and female **(D)** mice. **(E, F)** Differentially abundant bacterial taxa at the genus level in male **(E)** and female **(F)** fecal microbiota.

**Figure 6:**
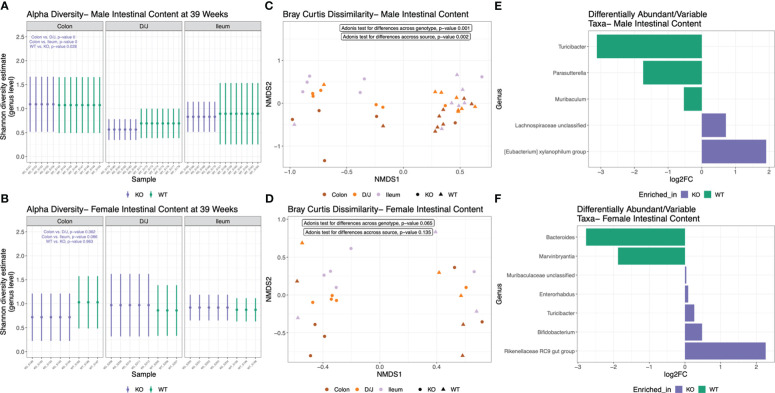
*Nlrp12* deficiency induces dynamic changes in intestinal microbiota diversity and composition. Intestinal microbiota was collected at the endpoint of 39 weeks of age. **(A, B)** Alpha diversity of intestinal microbiota based on Shannon diversity estimate in male **(A)** and female **(B)** B6/*lpr* mice upon alteration of NLRP12. **(C, D)** Non-metric multidimensional scaling showing the segregation of intestinal microbiota based on Bray Curtis dissimilarity of beta diversity in male **(C)** and female **(D)** mice. **(E, F)** Differentially abundant bacterial taxa at the genus level in male **(E)** and female **(F)** intestinal microbiota.

Changes of microbiota dynamics have been shown to drive autoimmunity (32, 33) or modulate autoimmunity (28). This has been established for SLE (37, 38) but not yet for ALPS. Interestingly, NLRP12 could shape inflammatory outcomes through regulating the gut microbiota (49, 85). Thus, we investigated whether the alteration of *Nlrp12* could implicate the gut microbiota in B6/*lpr* mice. We analyzed the fecal and intestinal microbiotas of both male and female mice seeking to answer the sex-dependent response to *Nlrp12* deficiency. We found a clear distinction in the fecal microbiota diversity with or without NLRP12 (**Figure 5**). Fecal microbiotas had significantly different alpha diversity on the genus level as shown by Shannon diversity estimate in both male (**Figure 5A**) and female (**Figure 5B**) B6/*lpr* mice, where *Nlrp12* deficiency led to significantly increased microbiota diversity. However, the difference in alpha diversity was much more pronounced in male than female mice. Similarly, analysis of beta diversity based on Bray Curtis dissimilarity calculation showed significantly different overall taxonomic composition based on the genotype but not the timepoint between WT and *Nlrp12^-/-^* B6/*lpr* male (**Figure 5C**) and female (**Figure 5D**) mice. Moreover, the composition of fecal microbiota changed upon alteration of *Nlrp12*. We detected significant enrichment of various genera in *Nlrp12^-/-^* B6/*lpr* male (**Figure 5E**) and female mice (**Figure 5F**). Strikingly, the intestinal microbiota diversity showed clear differences only in male mice that might explain the sex-dependent changes in disease phenotype. Analysis of alpha diversity from different intestinal segments (duodenum/jejunum, ileum, and colon) at 39 weeks of age showed that male (**Figure 6A**, P=0.028), but not female (**Figure 6B**, P=0.963), mice have distinct microbial composition upon alteration of *Nlrp12*. Similarly, the overall taxonomic composition was different for genotype and intestinal segment only among males (**Figure 6C**, P=0.001) but not females (**Figure 6D**, P=0.065). While not many changes were observed as in fecal microbiota, several genera were significantly altered in the intestinal microbiota of WT *vs. Nlrp12^-/-^* B6/*lpr* male (**Figure 6E**) and female mice (**Figure 6F**).

## Discussion

We investigated the role of NLRP12 in modulating autoimmune-associated inflammation utilizing the *Fas^lpr^* mutant mice as a model of ALPS and SLE (1). NLRP12 is an inhibitory checkpoint of inflammation; but at the same time, it can form inflammasome to promote inflammation (39). So far, triggers that direct the activity of NLRP12 to either way are not fully understood. The findings of this work support the hypothesis that NLRP12 can work towards the inflammasome activation pathway to deteriorate systemic autoimmunity. Inflammasome protein complex including NLRP12 has been proposed to be implicated in ALPS (86). Similarly, a recent study has shown that the expression of NLRP12 together with other inflammasome-forming innate sensors is increased in the SLE B cells (87). However, the mechanisms through which NLRP12 could modulate systemic autoimmunity are still elusive.

Here, we show how NLRP12 modulates cellular responses under autoimmune conditions. *Nlrp12* deficiency attenuated autoreactive B cell responses in B6/*lpr* mice, dampening production of autoantibodies and their renal deposition. Mechanistically, *Nlrp12* deficiency may have hindered terminal differentiation, GC formation, and survival of autoreactive B cells, suggesting B cells as a potential hub for NLRP12 inflammasome activity in autoimmune conditions. In parallel, NLRP12 is expressed at high levels in T cells (43) and has been shown to modulate the differentiation and responses of different T cell subsets (43, 54). Specifically, T cells can maintain an inflammatory milieu (88, 89) and potentiate B cell autoreactivity (72), a phenomenon implicated in both SLE (73) and ALPS (74–76). Interestingly, we found that NLRP12 could drive and maintain the accumulation of T cells in the spleen. The deficiency of *Nlrp12* reduced the percentage of splenic CD3^+^ T cells and importantly, the generation of DN-T cells and T_EM_ cells, which are known pathogenic T cell subsets in the autoimmunity (8, 74–76, 90). Moreover, our results suggest that NLRP12 might drive B cell activation through promoting Tfh cells, where mice with intact NLRP12 had an expansion of Tfh cells and upregulated levels of factors associated with B cell help (70). To this end, our findings warrant further investigation on the cell-specific mechanisms, either intrinsic or extrinsic, through which NLRP12 might target B cell autoreactivity to deteriorate systemic autoimmunity in male B6/*lpr* mice. Furthermore, we found decreased levels of inflammatory mediators including TNFα, MIP-3β, IL-1β and CCR7 in splenic tissues and/or BM-derived myeloid cell cultures following *ex-vivo* stimulation for *Nlrp12^-/-^* B6/*lpr* mice, supporting the notion that NLRP12 might trigger inflammasome activation in different immune cell populations to deteriorate systemic inflammation.

Importantly, *Nlrp12* deficiency dramatically altered the gut microbiota especially in male mice. Although *Nlrp12* alteration significantly changed the diversity and composition of fecal microbiota in both males and females, significant differences in the intestinal microbiota were seen only in male mice. This observation supports the notion that gut microbiota might drive the sex-dependent outcome of *Nlrp12* deficiency in our mouse model of systemic autoimmunity. However, future studies are still needed to mechanistically delineate our observations and to demonstrate the potential link between gut microbiota and the sex-dependent outcomes seen in *Nlrp12*-deficient mice. It is also likely that treating females with testosterone, or gut microbiota from male mice, will restore the male phenotype seen in this study.

In conclusion, the present study provides novel insight into the immunoregulatory role of NLRP12 in systemic autoimmune disorders such as ALPS and SLE. Attenuation of autoreactive cell responses including B, T, and myeloid cells that we have observed in the absence of NLRP12 supports a sex-dependent, pro-inflammatory role of NLRP12 under autoimmune conditions that warrant further investigation to decipher the underlying mechanisms. In addition, the marked differences in microbiota diversity and composition between WT and *Nlrp12^-/-^* B6/*lpr* mice suggest a microbiota-dependent role of NLRP12 in shaping autoimmune pathogenesis. Future studies will reveal a potential gut microbiota-dependent mechanism by which NLRP12 deficiency attenuates autoimmune pathologies in male mice. We will employ antibiotic treatment, co-housing, and gut microbiota transplantation experiments to determine whether changes of the gut microbiota are a cause, or an effect, of the attenuated disease phenotype in male *Nlrp12^-/-^* B6/*lpr* mice. As gut microbiota has been shown to drive autoimmunity in a sex-dependent manner (91), studies with mice deficient in androgen receptors will also reveal a potential role for male hormones that may work in concert with gut microbiota.

## Data availability statement

The data presented in the study are deposited in the NCBI repository, accession number PRJNA805257. https://www.ncbi.nlm.nih.gov/bioproject/PRJNA805257.

## Ethics statement

The animal study was reviewed and approved by IACUC of Virginia Tech.

## Author contributions

XL and SA conceived the study. IA provided B6.*Nlrp12^-/-^* mice. LA performed the research. JM, XC-P, JZ, JT and BS contributed to mouse sampling and tissue harvesting. ME contributed to mouse breeding. JSM analyzed the microbiota data. LA and XL analyzed the data and wrote the manuscript. SA edited the manuscript. All authors contributed to the article and approved the submitted version.

## References

[1] Cohen PL Eisenberg RA. Lpr and gld: single gene models of systemic autoimmunity and lymphoproliferative disease. Annu Rev Immunol (1991) 9(1):243–69. doi: 10.1146/annurev.iy.09.040191.001331 1910678

[2] Sneller MC Dale JK Straus SE. Autoimmune lymphoproliferative syndrome. Curr Opin Rheumatol (2003) 15(4):417–21. doi: 10.1097/00002281-200307000-00008 12819469

[3] Lockshin MD Barbhaiya M Izmirly P Buyon JP Crow MK. SLE: reconciling heterogeneity. Lupus Sci Med (2019) 6(1):e000280. doi: 10.1136/lupus-2018-000280 31080630 PMC6485210

[4] Tsokos GC Lo MS Reis PC Sullivan KE. New insights into the immunopathogenesis of systemic lupus erythematosus. Nat Rev Rheumatol (2016) 12(12):716–730. doi: 10.1038/nrrheum.2016.186 27872476

[5] Fisher GH Rosenberg FJ Straus SE Dale JK Middelton LA Lin AY. Dominant interfering fas gene mutations impair apoptosis in a human autoimmune lymphoproliferative syndrome. Cell (1995) 81(6):935–46. doi: 10.1016/0092-8674(95)90013-6 7540117

[6] Rieux-Laucat F Le Deist F Hivroz C Roberts I Debatin K Fischer A. Mutations in fas associated with human lymphoproliferative syndrome and autoimmunity. Science (1995) 268(5215):1347–9. doi: 10.1126/science.7539157 7539157

[7] White S Rosen A. Apoptosis in systemic lupus erythematosus. Curr Opin Rheumatol (2003) 15(5):557–62. doi: 10.1097/00002281-200309000-00006 12960480

[8] Crispín JC Oukka M Bayliss G Cohen RA Van Beek CA Stillman IE. Expanded double negative T cells in patients with systemic lupus erythematosus produce IL-17 and infiltrate the kidneys. J Immunol (2008) 181(12):8761–6. doi: 10.4049/jimmunol.181.12.8761 PMC2596652 19050297

[9] Mazerolles F Stolzenberg M-C Pelle O Picard C Neven B Fischer A. Autoimmune lymphoproliferative syndrome-FAS patients have an abnormal regulatory T cell (Treg) phenotype but display normal natural treg-suppressive function on T cell proliferation. Front Immunol (2018) 9:718. doi: 10.3389/fimmu.2018.00718 29686686 PMC5900038

[10] Teachey DT Manno CS Axsom KM Andrews T Choi JK Greenbaum BH. Unmasking Evans syndrome: T-cell phenotype and apoptotic response reveal autoimmune lymphoproliferative syndrome (ALPS). Blood (2005) 105(6):2443–8. doi: 10.1182/blood-2004-09-3542 15542578

[11] Fuss IJ Strober W Dale JK Fritz S Pearlstein GR Puck JM. Characteristic T helper 2 T cell cytokine abnormalities in autoimmune lymphoproliferative syndrome, a syndrome marked by defective apoptosis and humoral autoimmunity. J Immunol (1997) 158(4):1912–8. doi: 10.4049/jimmunol.158.4.1912 9029133

[12] Jackson CE Puck JM. Autoimmune lymphoproliferative syndrome, a disorder of apoptosis. Curr Opin Pediatr (1999) 11(6):521–7. doi: 10.1097/00008480-199912000-00009 10590910

[13] Ma C Xia Y Yang Q Zhao Y. The contribution of macrophages to systemic lupus erythematosus. Clin Immunol (2019) 207:1–9. doi: 10.1016/j.clim.2019.06.009 31255802

[14] Liao X Ren J Reihl A Pirapakaran T Sreekumar B Cecere T. Renal-infiltrating CD11c+ cells are pathogenic in murine lupus nephritis through promoting CD4+ T cell responses. Clin Exp Immunol (2017) 190(2):187–200. doi: 10.1111/cei.13017 28722110 PMC5629427

[15] Fiore N Castellano G Blasi A Capobianco C Loverre A Montinaro V. Immature myeloid and plasmacytoid dendritic cells infiltrate renal tubulointerstitium in patients with lupus nephritis. Mol Immunol (2008) 45(1):259–65. doi: 10.1016/j.molimm.2007.04.029 17570528

[16] Kaplan MJ. Neutrophils in the pathogenesis and manifestations of SLE. Nat Rev Rheumatol (2011) 7(12):691–9. doi: 10.1038/nrrheum.2011.132 PMC3243068 21947176

[17] Herrada AA Escobedo N Iruretagoyena M Valenzuela RA Burgos PI Cuitino L. Innate immune cells’ contribution to systemic lupus erythematosus. Front Immunol (2019) 10:772. doi: 10.3389/fimmu.2019.00772 31037070 PMC6476281

[18] Waldner H. The role of innate immune responses in autoimmune disease development. Autoimmun Rev (2009) 8(5):400–4. doi: 10.1016/j.autrev.2008.12.019 19162250

[19] Rönnblom L Pascual V. The innate immune system in SLE: Type I interferons and dendritic cells. Lupus (2008) 17(5):394–9. doi: 10.1177/0961203308090020 PMC3694565 18490415

[20] Moulton VR Tsokos GC. Abnormalities of T cell signaling in systemic lupus erythematosus. Arthritis Res Ther (2011) 13(2):1–10. doi: 10.1186/ar3251 PMC3132009 21457530

[21] Katsuyama T Tsokos GC Moulton VR. Aberrant T cell signaling and subsets in systemic lupus erythematosus. Front Immunol (2018) 9:1088. doi: 10.3389/fimmu.2018.01088 29868033 PMC5967272

[22] Kammer GM Perl A Richardson BC Tsokos GC. Abnormal T cell signal transduction in systemic lupus erythematosus. Arthritis Rheumatism (2002) 46(5):1139–54. doi: 10.1002/art.10192 12115215

[23] Morimoto C Reinherz EL Schlossman SF Schur P Mills J Steinberg A. Alterations in immunoregulatory T cell subsets in active systemic lupus erythematosus. J Clin Invest (1980) 66(5):1171–4. doi: 10.1172/JCI109948 PMC371557 7000827

[24] Lipsky PE. Systemic lupus erythematosus: An autoimmune disease of b cell hyperactivity. Nat Immunol (2001) 2(9):764–6. doi: 10.1038/ni0901-764 11526379

[25] Doerner T Jacobi AM Lee J Lipsky PE. Abnormalities of b cell subsets in patients with systemic lupus erythematosus. J Immunol Methods (2011) 363(2):187–97. doi: 10.1016/j.jim.2010.06.009 20598709

[26] Grammer AC Lipsky PE. B cell abnormalities in systemic lupus erythematosus. Arthritis Res Ther (2003) 5(4):1–6. doi: 10.1186/ar1009 PMC2833441 15180894

[27] Zharkova O Celhar T Cravens PD Satterthwaite AB Fairhurst A-M Davis LS. Pathways leading to an immunological disease: Systemic lupus erythematosus. Rheumatology (2017) 56(suppl_1):i55–66. doi: 10.1093/rheumatology/kew427 PMC5410978 28375453

[28] Yurkovetskiy LA Pickard JM Chervonsky AV. Microbiota and autoimmunity: Exploring new avenues. Cell Host Microbe (2015) 17(5):548–52. doi: 10.1016/j.chom.2015.04.010 PMC4535992 25974297

[29] Xu H Liu M Cao J Li X Fan D Xia Y. The dynamic interplay between the gut microbiota and autoimmune diseases. J Immunol Res (2019) 2019:7546047. doi: 10.1155/2019/7546047 31772949 PMC6854958

[30] Jiao Y Wu L Huntington ND Zhang X. Crosstalk between gut microbiota and innate immunity and its implication in autoimmune diseases. Front Immunol (2020) 11:282. doi: 10.3389/fimmu.2020.00282 32153586 PMC7047319

[31] López P de Paz B Rodríguez-Carrio J Hevia A Sánchez B Margolles A. Th17 responses and natural IgM antibodies are related to gut microbiota composition in systemic lupus erythematosus patients. Sci Rep (2016) 6(1):1–12. doi: 10.1038/srep24072 27044888 PMC4820712

[32] Zhang H Liao X Sparks JB Luo XM. Dynamics of gut microbiota in autoimmune lupus. Appl Environ Microbiol (2014) 80(24):7551–60. doi: 10.1128/AEM.02676-14 PMC4249226 25261516

[33] Luo XM Edwards MR Mu Q Yu Y Vieson MD Reilly CM. Gut microbiota in human systemic lupus erythematosus and a mouse model of lupus. Appl Environ Microbiol (2018) 84(4):e02288–17. doi: 10.1128/AEM.02288-17 PMC5795066 29196292

[34] Zhang L Qing P Yang H Wu Y Liu Y Luo Y. Gut microbiome and metabolites in systemic lupus erythematosus: Link, mechanisms and intervention. Front Immunol (2021) 12:686501. doi: 10.3389/fimmu.2021.686501 34335588 PMC8319742

[35] Ma Y Xu X Li M Cai J Wei Q Niu H. Gut microbiota promote the inflammatory response in the pathogenesis of systemic lupus erythematosus. Mol Med (2019) 25(1):1–16. doi: 10.1186/s10020-019-0102-5 31370803 PMC6676588

[36] Rosser EC Mauri C. A clinical update on the significance of the gut microbiota in systemic autoimmunity. J Autoimmun (2016) 74:85–93. doi: 10.1016/j.jaut.2016.06.009 27481556

[37] Mu Q Zhang H Liao X Lin K Liu H Edwards MR. Control of lupus nephritis by changes of gut microbiota. Microbiome (2017) 5(1):1–12. doi: 10.1186/s40168-017-0300-8 28697806 PMC5505136

[38] Mu Q Edwards MR Swartwout BK Cabana Puig X Mao J Zhu J. Gut microbiota and bacterial DNA suppress autoimmunity by stimulating regulatory b cells in a murine model of lupus. Front Immunol (2020) 11:2911. doi: 10.3389/fimmu.2020.593353 PMC7683516 33240280

[39] Tuladhar S Kanneganti T-D. NLRP12 in innate immunity and inflammation. Mol Aspects Med (2020), 76:100887. doi: 10.1016/j.mam.2020.100887 32838963 PMC9375713

[40] Lich JD Williams KL Moore CB Arthur JC Davis BK Taxman DJ. Cutting edge: Monarch-1 suppresses non-canonical NF-κB activation and p52-dependent chemokine expression in monocytes. J Immunol (2007) 178(3):1256–60. doi: 10.4049/jimmunol.178.3.1256 17237370

[41] Zaki MH Vogel P Malireddi RS Body-Malapel M Anand PK Bertin J. The NOD-like receptor NLRP12 attenuates colon inflammation and tumorigenesis. Cancer Cell (2011) 20(5):649–60. doi: 10.1016/j.ccr.2011.10.022 PMC3761879 22094258

[42] Arthur JC Lich JD Ye Z Allen IC Gris D Wilson JE. Cutting edge: NLRP12 controls dendritic and myeloid cell migration to affect contact hypersensitivity. J Immunol (2010) 185(8):4515–9. doi: 10.4049/jimmunol.1002227 PMC3641837 20861349

[43] Lukens JR Gurung P Shaw PJ Barr MJ Zaki MH Brown SA. The NLRP12 sensor negatively regulates autoinflammatory disease by modulating interleukin-4 production in T cells. Immunity (2015) 42(4):654–64. doi: 10.1016/j.immuni.2015.03.006 PMC4412374 25888258

[44] Thaiss CA Elinav E. NF-κB regulation by NLRs: T cells join the club. Immunity (2015) 42(4):595–7. doi: 10.1016/j.immuni.2015.03.010 25902475

[45] Williams KL Taxman DJ Linhoff MW Reed W Ting JP-Y. Cutting edge: Monarch-1: a pyrin/nucleotide-binding domain/leucine-rich repeat protein that controls classical and nonclassical MHC class I genes. J Immunol (2003) 170(11):5354–8. doi: 10.4049/jimmunol.170.11.5354 12759408

[46] Ulland TK Jain N Hornick EE Elliott EI Clay GM Sadler JJ. Nlrp12 mutation causes C57BL/6J strain-specific defect in neutrophil recruitment. Nat Commun (2016) 7(1):1–13. doi: 10.1038/ncomms13180 PMC5093323 27779193

[47] Hornick EE Banoth B Miller AM Zacharias ZR Jain N Wilson ME. Nlrp12 mediates adverse neutrophil recruitment during influenza virus infection. J Immunol (2018) 200(3):1188–97. doi: 10.4049/jimmunol.1700999 PMC5831365 29282312

[48] Prado DS Veras FP Ferreira RG Damasceno LEA Melo PH Zamboni DS. NLRP12 controls arthritis severity by acting as a checkpoint inhibitor of Th17 cell differentiation. FASEB J (2020) 34(8):10907–19. doi: 10.1096/fj.202000795R 32632939

[49] Truax AD Chen L Tam JW Cheng N Guo H Koblansky AA. The inhibitory innate immune sensor NLRP12 maintains a threshold against obesity by regulating gut microbiota homeostasis. Cell Host Microbe (2018) 24(3):364–78.e6. doi: 10.1016/j.chom.2018.08.009 30212649 PMC6161752

[50] Sun Z Pei W Guo Y Wang Z Shi R Chen X. Gut microbiota-mediated NLRP12 expression drives the attenuation of dextran sulphate sodium-induced ulcerative colitis by qingchang wenzhong decoction. Evidence-Based Complementary Altern Med (2019) 2019:9839474. doi: 10.1155/2019/9839474 PMC6466890 31061672

[51] Chen L Wilson JE Koenigsknecht MJ Chou W-C Montgomery SA Truax AD. NLRP12 attenuates colon inflammation by maintaining colonic microbial diversity and promoting protective commensal bacterial growth. Nat Immunol (2017) 18(5):541–51. doi: 10.1038/ni.3690 PMC5395345 28288099

[52] Allen IC Wilson JE Schneider M Lich JD Roberts RA Arthur JC. NLRP12 suppresses colon inflammation and tumorigenesis through the negative regulation of noncanonical NF-κB signaling. Immunity (2012) 36(5):742–54. doi: 10.1016/j.immuni.2012.03.012 PMC3658309 22503542

[53] Gharagozloo M Mahvelati TM Imbeault E Gris P Zerif E Bobbala D. The nod-like receptor, Nlrp12, plays an anti-inflammatory role in experimental autoimmune encephalomyelitis. J Neuroinflamm (2015) 12(1):1–13. doi: 10.1186/s12974-015-0414-5 PMC4628289 26521018

[54] Gharagozloo M Mahmoud S Simard C Mahvelati TM Amrani A Gris D. The dual immunoregulatory function of Nlrp12 in T cell-mediated immune response: Lessons from experimental autoimmune encephalomyelitis. Cells (2018) 7(9):119. doi: 10.3390/cells7090119 30150571 PMC6162721

[55] Abdelhamid L Cabana-Puig X Swartwout B Lee J Li S Sun S. Retinoic acid exerts disease stage-dependent effects on pristane-induced lupus. Front Immunol (2020) 11:408(408). doi: 10.3389/fimmu.2020.00408 32265909 PMC7103630

[56] Goodridge HS Shimada T Wolf AJ Hsu Y-MS Becker CA Lin X. Differential use of CARD9 by dectin-1 in macrophages and dendritic cells. J Immunol (2009) 182(2):1146–54. doi: 10.4049/jimmunol.182.2.1146 PMC2718573 19124758

[57] Abdelhamid L Cabana-Puig X Mu Q Moarefian M Swartwout B Eden K. Quaternary ammonium compound disinfectants reduce lupus-associated splenomegaly by targeting neutrophil migration and T-cell fate. Front Immunol (2020) 11:2738. doi: 10.3389/fimmu.2020.575179 PMC7609861 33193366

[58] Ley RE Bäckhed F Turnbaugh P Lozupone CA Knight RD Gordon JI. Obesity alters gut microbial ecology. Proc Natl Acad Sci (2005) 102(31):11070–5. doi: 10.1073/pnas.0504978102 PMC1176910 16033867

[59] Caporaso JG Lauber CL Walters WA Berg-Lyons D Huntley J Fierer N. Ultra-high-throughput microbial community analysis on the illumina HiSeq and MiSeq platforms. ISME J (2012) 6(8):1621–4. doi: 10.1038/ismej.2012.8 PMC3400413 22402401

[60] McMurdie PJ Holmes S. Phyloseq: An r package for reproducible interactive analysis and graphics of microbiome census data. PloS One (2013) 8(4):e61217. doi: 10.1371/journal.pone.0061217 23630581 PMC3632530

[61] Callahan BJ McMurdie PJ Rosen MJ Han AW Johnson AJA Holmes SP. DADA2: High-resolution sample inference from illumina amplicon data. Nat Methods (2016) 13(7):581–3. doi: 10.1038/nmeth.3869 PMC4927377 27214047

[62] McLaren. M. mikemc/dada2-reference-databases: Silva 138.1 (Version v2). Zenodo (2021). doi: 10.5281/zenodo.4587946

[63] Martin BD Witten D Willis AD. Modeling microbial abundances and dysbiosis with beta-binomial regression. Ann Appl Stat (2020) 14(1):94. doi: 10.1214/19-AOAS1283 32983313 PMC7514055

[64] Willis AD Martin BD. Estimating diversity in networked ecological communities. Biostatistics (2020) 23(1):207–222. doi: 10.1093/biostatistics/kxaa015 PMC8759443 32432696

[65] Ngo ST Steyn FJ McCombe PA. Gender differences in autoimmune disease. Front Neuroendocrinol (2014) 35(3):347–69. doi: 10.1016/j.yfrne.2014.04.004 24793874

[66] Munoz-Grajales C Gonzalez L Alarcon G Acosta-Reyes J. Gender differences in disease activity and clinical features in newly diagnosed systemic lupus erythematosus patients. Lupus (2016) 25(11):1217–23. doi: 10.1177/0961203316635286 26921269

[67] Wasef SZY. Gender differences in systemic lupus erythematosus. Gender Med (2004) 1(1):12–7. doi: 10.1016/S1550-8579(04)80006-8 16115579

[68] Macedo E Appenzeller S Costallat L. Gender differences in systemic lupus erythematosus concerning anxiety, depression and quality of life. Lupus (2016) 25(12):1315–27. doi: 10.1177/0961203316638934 26989166

[69] Bélanger S Crotty S. Dances with cytokines, featuring TFH cells, IL-21, IL-4 and b cells. Nat Immunol (2016) 17(10):1135–6. doi: 10.1038/ni.3561 27648538

[70] Rao DA. T Cells that help b cells in chronically inflamed tissues. Front Immunol (2018) 9:1924(1924). doi: 10.3389/fimmu.2018.01924 30190721 PMC6115497

[71] Gris D Ye Z Iocca HA Wen H Craven RR Gris P. NLRP3 plays a critical role in the development of experimental autoimmune encephalomyelitis by mediating Th1 and Th17 responses. J Immunol (2010) 185(2):974–81. doi: 10.4049/jimmunol.0904145 PMC3593010 20574004

[72] Mountz JD Hsu H-C Ballesteros-Tato A. Dysregulation of T follicular helper cells in lupus. J Immunol (2019) 202(6):1649–58. doi: 10.4049/jimmunol.1801150 PMC6402788 30833421

[73] Suárez-Fueyo A Bradley SJ Tsokos GC. T Cells in systemic lupus erythematosus. Curr Opin Immunol (2016) 43:32–8. doi: 10.1016/j.coi.2016.09.001 PMC5125867 27636649

[74] Bristeau-Leprince A Mateo V Lim A Magerus-Chatinet A Solary E Fischer A. Human TCR α/β+ CD4– CD8– double-negative T cells in patients with autoimmune lymphoproliferative syndrome express restricted vβ TCR diversity and are clonally related to CD8+ T cells. J Immunol (2008) 181(1):440–8. doi: 10.4049/jimmunol.181.1.440 18566410

[75] Rieux-Laucat F Magerus-Chatinet A. Autoimmune lymphoproliferative syndrome: a multifactorial disorder. Haematologica (2010) 95(11):1805–7. doi: 10.3324/haematol.2010.030395 PMC2966899 21037326

[76] Bleesing JJ Brown MR Novicio C Guarraia D Dale JK Straus SE. A composite picture of TcR alpha/beta(+) CD4(-)CD8(-) T cells (alpha/beta-DNTCs) in humans with autoimmune lymphoproliferative syndrome. Clin Immunol (2002) 104(1):21–30. doi: 10.1006/clim.2002.5225 12139944

[77] Crispín JC Tsokos GC. Human TCR-αβ+ CD4– CD8– T cells can derive from CD8+ T cells and display an inflammatory effector phenotype. J Immunol (2009) 183(7):4675–81. doi: 10.4049/jimmunol.0901533 PMC2878279 19734235

[78] Mehal WZ Crispe IN. TCR ligation on CD8+ T cells creates double-negative cells *in vivo* . J Immunol (1998) 161(4):1686–93. doi: 10.4049/jimmunol.161.4.1686 9712032

[79] Rodríguez-Rodríguez N Apostolidis SA Penaloza-MacMaster P Villa JMM Barouch DH Tsokos GC. Programmed cell death 1 and Helios distinguish TCR-αβ+ double-negative (CD4– CD8–) T cells that derive from self-reactive CD8 T cells. J Immunol (2015) 194(9):4207–14. doi: 10.4049/jimmunol.1402775 PMC4503929 25825451

[80] Meng F Lowell CA. Lipopolysaccharide (LPS)-induced macrophage activation and signal transduction in the absence of src-family kinases hck, fgr, and Lyn. J Exp Med (1997) 185(9):1661–70. doi: 10.1084/jem.185.9.1661 PMC2196288 9151903

[81] Abdi K Singh NJ Matzinger P. Lipopolysaccharide-activated dendritic cells:”exhausted” or alert and waiting? J Immunol (2012) 188(12):5981–9. doi: 10.4049/jimmunol.1102868 PMC3370068 22561154

[82] Riol-Blanco L Sánchez-Sánchez N Torres A Tejedor A Narumiya S Corbí AL. The chemokine receptor CCR7 activates in dendritic cells two signaling modules that independently regulate chemotaxis and migratory speed. J Immunol (2005) 174(7):4070–80. doi: 10.4049/jimmunol.174.7.4070 15778365

[83] Dinarello CA. A clinical perspective of IL-1β as the gatekeeper of inflammation. Eur J Immunol (2011) 41(5):1203–17. doi: 10.1002/eji.201141550 21523780

[84] Maurer M Von Stebut E. Macrophage inflammatory protein-1. Int J Biochem Cell Biol (2004) 36(10):1882–6. doi: 10.1016/j.biocel.2003.10.019 15203102

[85] Ray K. NLRP12 regulates gut microbiota to suppress intestinal inflammation. Nat Rev Gastroenterol Hepatol (2017) 14(5):261–. doi: 10.1038/nrgastro.2017.43 28356582

[86] Palmisani E Miano M Grossi A Lanciotti M Lupia M Terranova P. Autoimmune lymphoproliferative syndrome (ALPS) disease and ALPS phenotype: Are they two distinct entities? Hemasphere (2023) 7(3):e845. doi: 10.1097/HS9.0000000000000845 36844186 PMC9949771

[87] Yang M Long D Hu L Zhao Z Li Q Guo Y. AIM2 deficiency in b cells ameliorates systemic lupus erythematosus by regulating Blimp-1–Bcl-6 axis-mediated b-cell differentiation. Signal Transduction Targeted Ther (2021) 6(1):341. doi: 10.1038/s41392-021-00725-x PMC8440614 34521812

[88] Koga T Hedrich CM Mizui M Yoshida N Otomo K Lieberman LA. CaMK4-dependent activation of AKT/mTOR and CREM-α underlies autoimmunity-associated Th17 imbalance. J Clin Invest (2014) 124(5):2234–45. doi: 10.1172/JCI73411 PMC4001553 24667640

[89] Zhou H Li B Li J Wu T Jin X Yuan R. Dysregulated T cell activation and aberrant cytokine expression profile in systemic lupus erythematosus. Mediators Inflammation (2019) 2019:8450947. doi: 10.1155/2019/8450947 PMC6441516 31007604

[90] Devarajan P Chen Z. Autoimmune effector memory T cells: The bad and the good. Immunol Res (2013) 57(1-3):12–22. doi: 10.1007/s12026-013-8448-1 24203440 PMC4067599

[91] Markle JG Frank DN Mortin-Toth S Robertson CE Feazel LM Rolle-Kampczyk U. Sex differences in the gut microbiome drive hormone-dependent regulation of autoimmunity. Science (2013) 339(6123):1084–8. doi: 10.1126/science.1233521 23328391

