# Supplementary material for: Nlrp12 deficiency alters gut microbiota and ameliorates Faslpr -mediated systemic autoimmunity in male mice
Source: Front Immunol. 2023 Mar 10;14:1120958. doi: 10.3389/fimmu.2023.1120958 (PMC10036793; doi:10.3389/fimmu.2023.1120958)
Supplement: Supplementary file 1 [file DataSheet_1.pdf]

Supplemental Information

Table S1. Mouse primer sequences.

**Target**	**Forward Sequence**	**Reverse Sequence**
**18S rRNA**	GTAACCCGTTGAACCCCATT	CCATCCAATCGGTAGTAGCG
Bcl6	CACACCCGTCCATCATTGAA	TGTCCTCACGGTGCCTTTTT
Blimp1	GAGTACATACCGAAGGGAACA	CATCAATGAAGTGGTGGAAC
***Tnfsf13b*/BAFF**	ACACTGCCCAACAATTCCTG	TCGTCTCCGTTGGGTGAAATC
Il21	GGACCCTTGTCTGTCTGGTAG	TGTGGAGCTGATAGAAGTTCAGG
Tnf	CCCTCACACTCAGATCATCTTCT	GCTACGACGTGGGCTACAG
Il1β	GCAACTGTTCCTGAACTCAACT	CAGGCTGTCTTTTGTCAACGA
Il4	GGTCTCAACCCCCAGCTAGT	GCCGATCTCTCTCAAGTGAT
Cxcl13	GAGGCAGATGGAACTTGAGC	CTGGGGATCTTCGAATGCTA
***Ccl19*/MIP-3β**	CCTGGGAACATCGTGAAGC	TAGTGTGGTGAACACAACAGC
Ccr7	TCATTGCCGTGGTGGTAGTCTTCA	ATGTTGAGCTGCTTGCTGGTTTCG
Androgen Receptor	GATGGTATTTGCCATGGGTTG	GGCTGTACATCCGAGACTTGTG

A

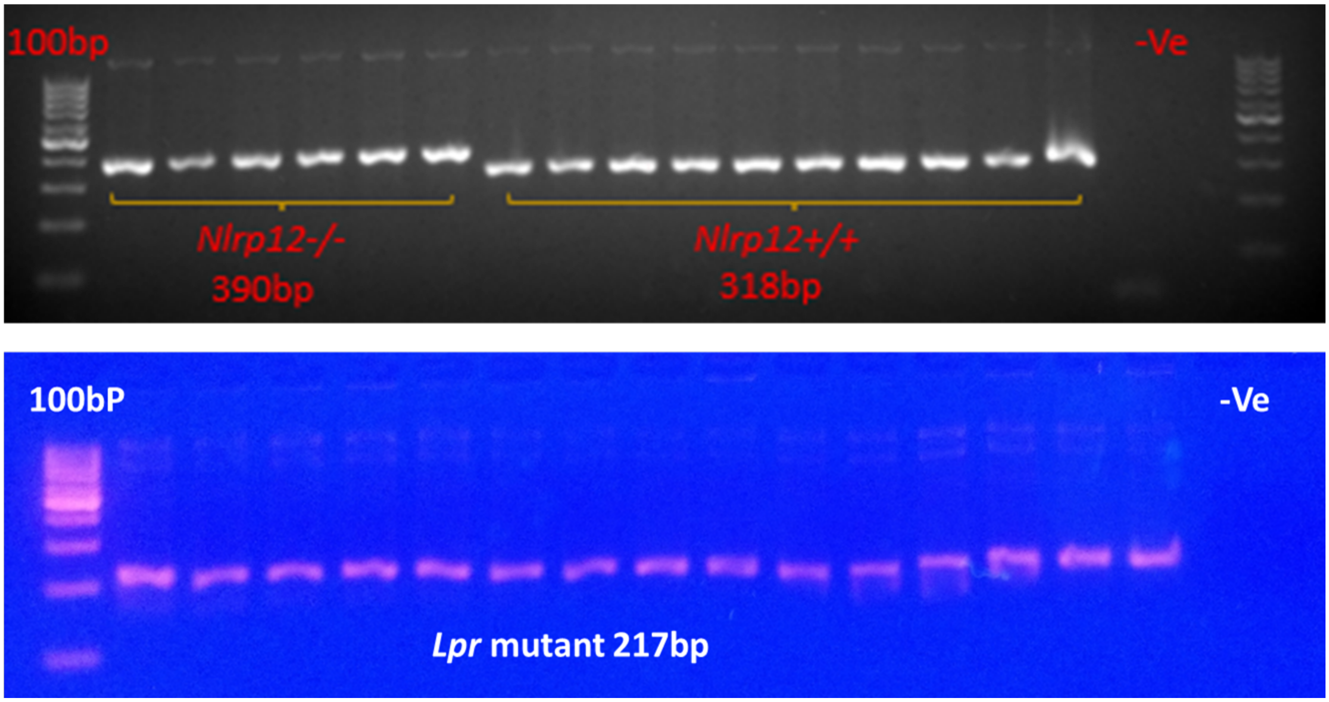

Figure S1. Representative gel analysis of PCR genotyping products for both *Nlrp12* (upper; *Nlrp12*$^{-/-}$ at 390 bp and *Nlrp12*$^{+/+}$ at 318 bp) and *Fas*$^{lpr/lpr}$ mutant (lower; 217 bp).

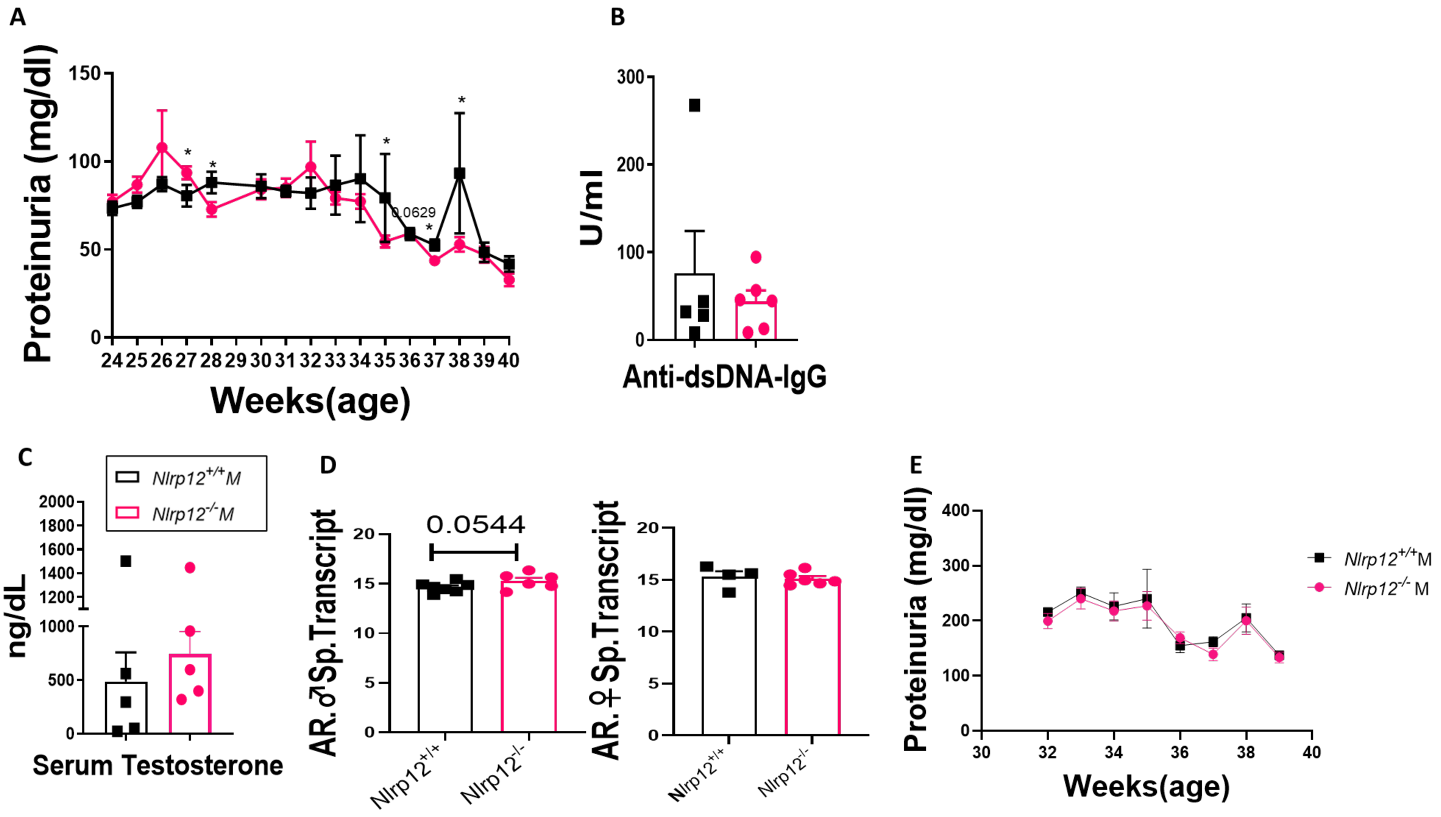

Figure S2. (A) Level of proteinuria over time in female mice (n=6 or 14/group). (B) Level of anti-dsDNA IgG antibodies in female serum at 39 weeks of age. (C) Level of serum testosterone in male mice. (D) Splenic transcript level of androgen receptor in male (left) and female mice (right). (E) Levels of proteinuria beyond 32 weeks of age in male mice (n=6 or 8/group). Data are shown as mean ± SEM.

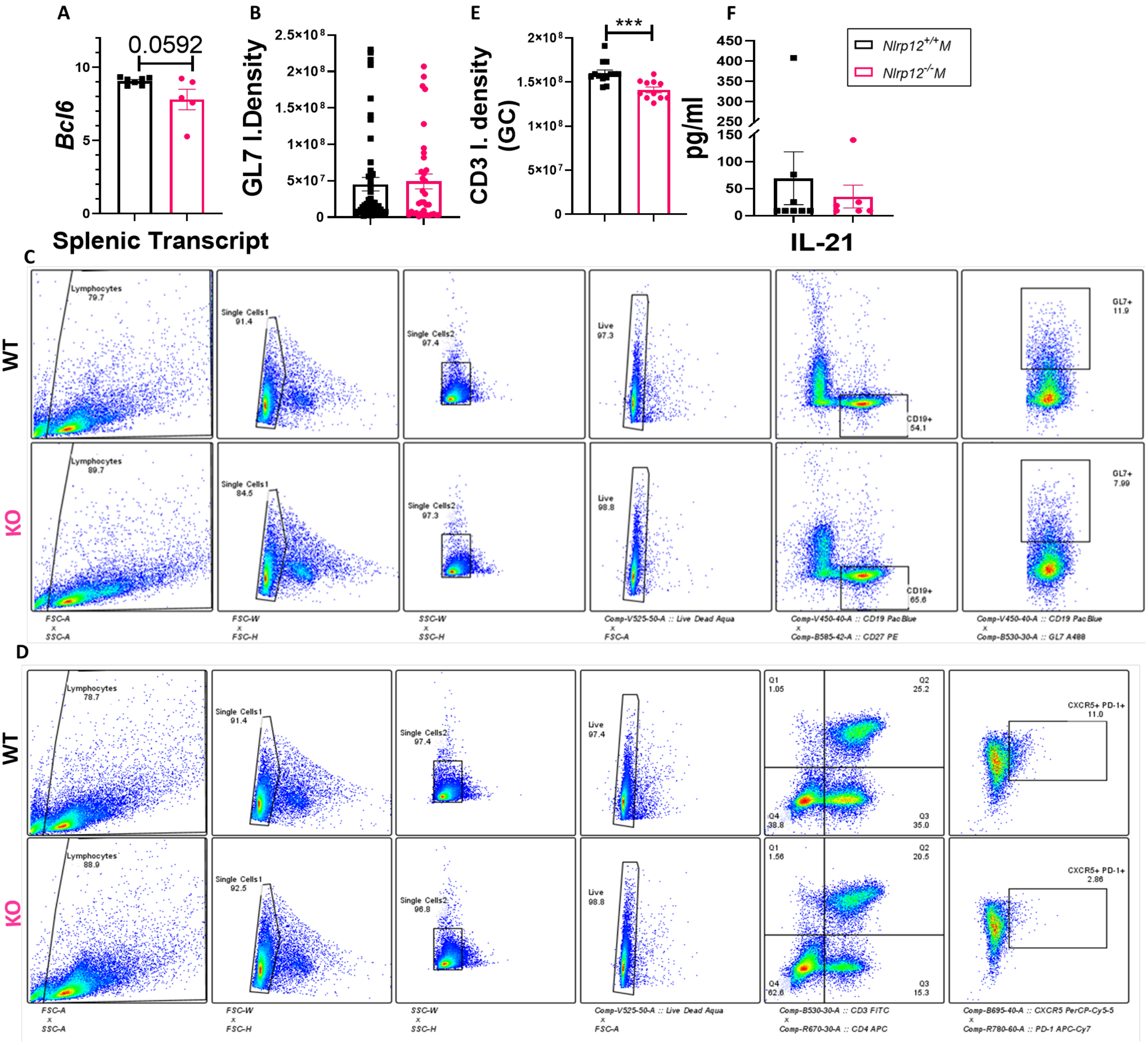

Figure S3. (A) Relative transcript level of splenic *Bcl6.* (B) The integrated density scores of GL7-FITC in immunohistochemical stains of splenic sections as quantified using ImageJ. n=3-4 per group, and the integrated density score was measured from the whole image. (C) Representative flow cytometry plots showing the gating strategy for GL7^{+} splenic CD19^{+} B lymphocytes. The last graphs on the right were pre-gated on CD19^{+} B cells. (D) Representative flow cytometry plots showing the gating strategy for CXCR5^{+}PD-1^{+}CD4^{+}CD3^{+} splenic Tfh cells. The last graphs on the right were pre-gated on CD3^{+}CD4^{+} T cells. (E) The integrated density scores of CD3-APC in immunohistochemical stains of splenic sections as quantified using ImageJ. n=3-4 per group, and multiple areas were quantified from each image as there were both dim and bright regions. (F) Level of serum IL-21 as determined with Luminex assay. Data are shown as mean ± SEM.

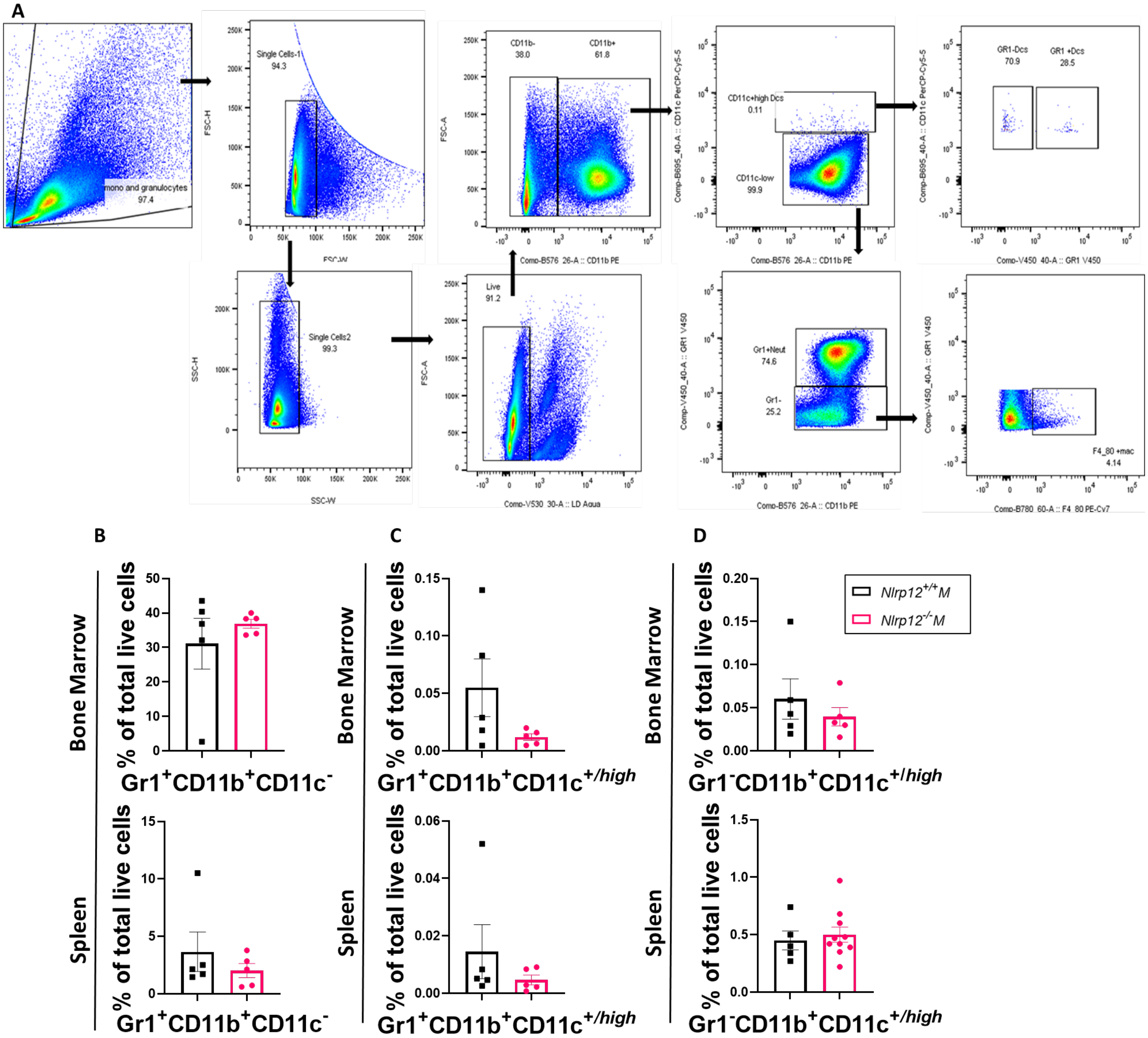

Figure S4. (A) Gating strategy for myeloid cells including dendritic cells (DCs) as $CD11c^{high}CD11b^{+}Gr1^{-}$ or $CD11c^{high}CD11b^{+}Gr1^{+}$, neutrophils as $CD11c^{-}CD11b^{+}Gr1^{+}$, and macrophages as $CD11c^{-/low}CD11b^{+}Gr1^{-}F4/80^{+}$. (B-D) The percentages of neutrophils (B), $Gr1^{+}$ DCs (C), and $Gr1^{-}$ DCs (D) in total bone marrow (upper panels) or splenic (lower panels) cells. Data are shown as mean ± SEM.

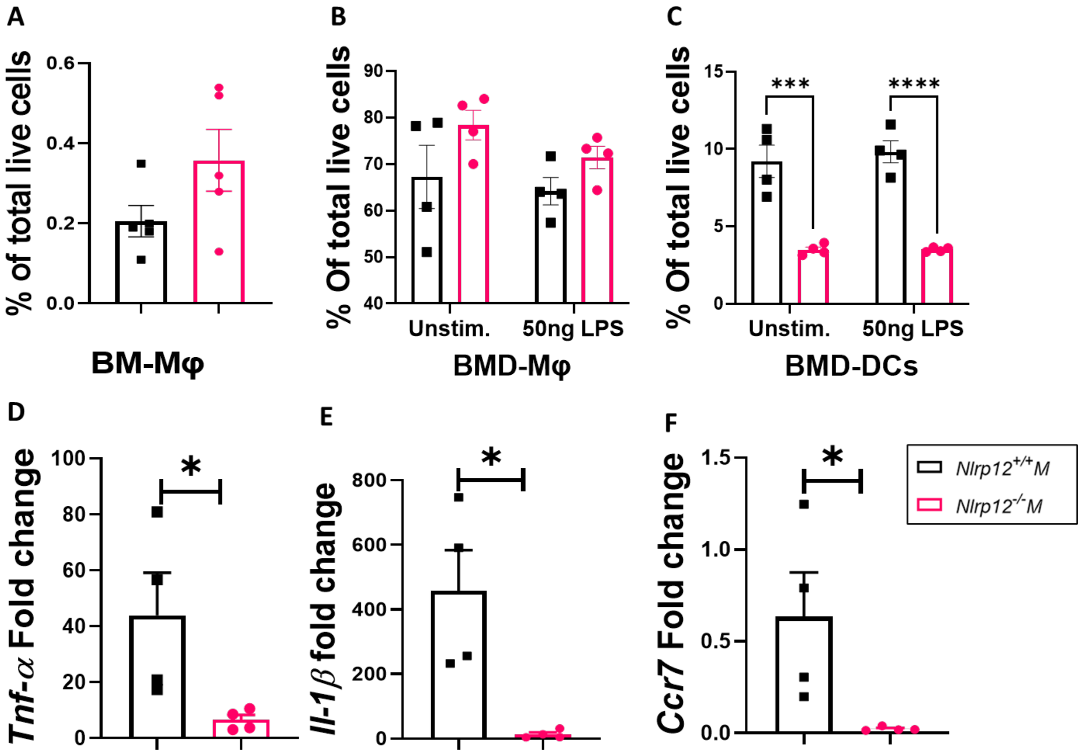

Figure S5. (A) Percentage of BM macrophages. (B-C) Percentage of Gr1$^-$CD11b$^+$CD11c$^-$ bone marrow-derived (BMD) macrophages (B) and Gr1$^-$CD11c$^+$CD11b$^+$ BMD-DCs (C) with or without 50 ng/ml LPS stimulation for 4 h. (D-F) Transcript levels of *Tnf* (D), *Il1β* (E) and *Ccr7* (F) following 4-h stimulation with 1 µg/ml LPS. Relative transcript quantities were calculated using the 2-ΔΔCt method and normalized to the level of the 18S rRNA housekeeping gene. ΔCt levels are shown. Data are shown as mean ± SEM.
